# Prevalence of Malocclusion Among School Children in Bangalore, India

**DOI:** 10.5005/jp-journals-10005-1002

**Published:** 2008-12-26

**Authors:** Usha Mohan Das, Venkatsubramanian, Divya Reddy

**Affiliations:** 1 President, Professor and Head, Department of Pedodontics and Preventive Dentistry, VS Dental College and Hospital, Bangalore, Karnataka, India; 2 Senior Lecturer, Department of Pedodontics and Preventive Dentistry, VS Dental College and Hospital, Bangalore, Karnataka, India; 3 Postgraduate, Department of Pedodontics and Preventive Dentistry, VS Dental College and Hospital, Bangalore, Karnataka, India

**Keywords:** Prevalence, Malocclusion, India.

## Abstract

The objective of this study was to determine the prevalence of malocclusion among school children of Bangalore city, India during their mixed dentition period. The sample consisted of 745 children (388 males and 357 females) in the age group of 8-12 years randomly selected from twelve different schools in Bangalore city. The subjects were randomly selected, and none had received previous orthodontic treatment. Occlusal anteroposterior relationships were assessed based on the Angle classification. Also various malocclusion features associated with class I malocclusion according to Dewey’s modification of Angle’s classification were assessed. The results showed that about 71% of the subjects had malocclusion. Class I malocclusion constituted the major proportion of malocclusion which was found in 62% of the studied population. No significant difference was found between boys and girls neither in the overall prevalence of malocclusion nor in various forms of malocclusion. Crowded incisors was found to be most ommon finding in subjects with class I malocclusion. A number of studies have been conducted to determine the prevalence of malocclusion among Indian children and it has been reported that the results range from a value as low as 19.6% (Miglani DC, Chennai 1961) to as high as 90% (Sidhu SS, Delhi). This varied range emphasizes the need to standardize criteria for assessing malocclusion.

## INTRODUCTION

Well aligned teeth not only contribute to the health of the oral cavity and stomatognathic system, but also influence the personality of the individual. Malocclusion compromises the health of oral tissues and also can lead to psychological and social problems.

A systematic and well-organized dental care program for any target population in a community requires some basic information, such as the prevalence of the condition. In more developed parts of the world, where the specialties of Orthodontics and Pedodontics have been established, adequate basic information is available^1^-^8^ on the prevalence of this condition. In developing nations, such information still lack.^9^

With increasing interest in the early detection and treatment of malocclusion and a corresponding emphasis on preventive procedures, it would be beneficial to collect more information on patients at younger age levels.

Therefore, the aim of this study is to estimate the prevalence of malocclusion in school children of Bangalore city, India during their mixed dentition period so as to take preventive measures to recognize and minimize the potential irregularities in the developing dentofacial complex.

## MATERIALS AND METHODS

The sample consisted of 745 children (388 males and 357 females) in the age group of 8-12 years randomly selected from twelve different schools in Bangalore city. None of the subjects had previous orthodontic treatment, and all had their first permanent molars.

All the students were examined by a single operator after obtaining the informed consent from the subjects and their parents. Approval to conduct the study was also received from the appropriate school authority.

The students were examined at their respective schools, using sterile mouth mirror and flash light.

All occlusal relationships were evaluated at a centric occlusion position, which was achieved by asking the subject to swallow and then to bite on his or her teeth together. The occlusion was then classified into normal occlusion or malocclusion using the first permanent molars as described by Angle. The cheeks were fully retracted to obtain a direct lateral view of the dentition in occlusion on each side.

Children with class I molar relationship, minimal overbite and overjet, proper alignment, and minimal crowding were classified as normal.

In subjects with class I malocclusion, class I molar relation existed with one or more of these characteristics: crowded incisors or labial canines, or both (Dewey type I), protruded maxillary incisors (Dewey type II), anterior end to end occlusion or anterior cross bite or both (Dewey type III), unilateral or bilateral posterior cross bite (Dewey type IV), mesial drift of molars (Dewey type V), anterior or posterior open bite, deep anterior overbite. The prevalence of these features were assessed in subjects showing Class I malocclusion.

The collected data were tabulated and analyzed statistically.

## RESULTS

**Fig. 1:**
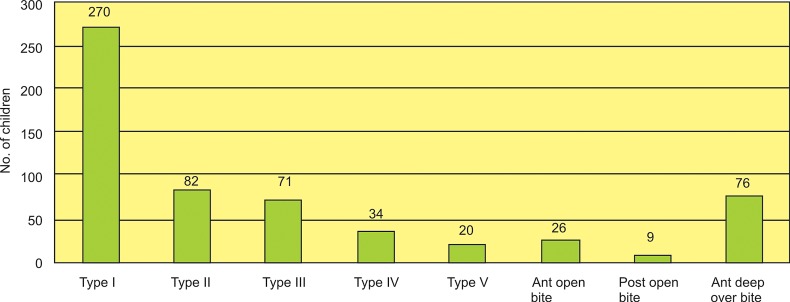
Distribution of types of class I malocclusion according to Dewey’s modification

Table 1 shows the occlusal classifications of the subjects. Normal occlusions were found in 29% of subjects, and 71% had malocclusions. Figure 1 shows the distribution of various types of class I malocclusion according to Dewey’s modification. Table 2 shows the gender distribution of normal occlusion along with various forms of malocclusions. No statistically significant relationship was found for any gender variation as tested by chi-square test by taking P values of less than 0.05 as statistically significant.

**Table 1:** TABLE 1: Occlusal classifications

Occlusal classification		N		%
Normal occlusion		219		29.4
Class I		459		61.6
Class II division I		51		6.8
Class II division II		12		1.6
Class III		4		0.6
**Total**		**745**		**100**

**Table 2:** TABLE 2: Gender distribution of occlusal variations

*Occlusion*		*Male(n)*		*Female(n)*
Normal occlusion		102		117
Class I		238		221
Class II division I		33		18
Class II division II		6		6
Class III		4		0
**Total**		**383**		**362**

## DISCUSSION

The results showed that 71% of the school children surveyed had malocclusion. This is similar to the findings of Prasad A Rajendra and Savadi S^10^ who conducted an epidemiological study of malocclusion in the age group of 5-15 years in Bangalore city in 1971, reported a high incidence of malocclusion of 85.7% with 51.5% class I, 4% class II, 0.9% class III.

Findings of this present study are in disagreement with Nagaraja Rao (1980)^10^ who found only 28.8% prevalence of malocclusion in school children of Udipi, Karnataka.

Class I malocclusion constituted the major proportion of malocclusion which was found in 62% of the studied population which is in agreement with the other studies.

No significant difference was found between boys and girls neither in the overall prevalence of malocclusion nor in various forms of malocclusion.

The present study evaluated various malocclusion features associated with class I malocclusion according to Dewey’s modification of Angle’s classification.

Crowded incisors was found to be most common finding in subjects with class I malocclusion followed by protruded maxillary incisors, anterior deep overbite, anterior cross bite, posterior cross bite and mesial drift of molars in descending order.

## CONCLUSION

The following conclusions are drawn from the present study:

- Prevalence of malocclusion was found to 71%.
- Class I malocclusion is the most prevalent occlusal pattern.
- Crowded incisors were the most common feature associated with class I malocclusion.
- No statistically significant sex differences were found among the subjects.

A number of studies have been conducted to determine the prevalence of malocclusion among Indian children and it has been reported that the results range from a value as low as 19.6% (Miglani DC, Chennai 1961) to as high as 90% (Sidhu SS, Delhi). This varied range emphasizes the need to standardize criteria for assessing malocclusion.
